# Evolution of the Proto Sex-Chromosome in *Solea senegalensis*

**DOI:** 10.3390/ijms20205111

**Published:** 2019-10-15

**Authors:** María Esther Rodríguez, Belén Molina, Manuel Alejandro Merlo, Alberto Arias-Pérez, Silvia Portela-Bens, Aglaya García-Angulo, Ismael Cross, Thomas Liehr, Laureana Rebordinos

**Affiliations:** 1 Área de Genética, Facultad de Ciencias del Mar y Ambientales, INMAR, Universidad de Cádiz, 11510 Cádiz, Spain; mariaesther.rodriguez@uca.es (M.E.R.); belen.molinafe@alum.uca.es (B.M.); alejandro.merlo@uca.es (M.A.M.); alberto.arias@uca.es (A.A.-P.); silvia.portela@uca.es (S.P.-B.); aglaya.garcia@uca.es (A.G.-A.); ismael.cross@uca.es (I.C.); 2 Molcular Cytogenetics, University Clinic Jena Institute of Human Genetics, 07747 Jena, Germany; thomas.liehr@med.uni-jena.de

**Keywords:** *Solea senegalensis*, genomic synteny, repetitive sequences, chromosome evolution, sex chromosomes, karyotype

## Abstract

*Solea senegalensis* is a flatfish belonging to the Soleidae family within the Pleuronectiformes order. It has a karyotype of 2*n* = 42 (FN = 60; 6M + 4 SM + 8 St + 24 T) and a XX/XY system. The first pair of metacentric chromosomes has been proposed as a proto sex-chromosome originated by a Robertsonian fusion between acrocentric chromosomes. In order to elucidate a possible evolutionary origin of this chromosome 1, studies of genomic synteny were carried out with eight fish species. A total of 88 genes annotated within of 14 BACs located in the chromosome 1 of *S. senegalensis* were used to elaborate syntenic maps. Six BACs (BAC5K5, BAC52C17, BAC53B20, BAC84K7, BAC56H24, and BAC48P7) were distributed in, at least, 5 chromosomes in the species studied, and a group of four genes from BAC53B20 (*grsf1*, *rufy3*, *slc4a4* and *npffr2*) and genes from BAC48K7 (*dmrt2*, *dmrt3*, *dmrt1*, *c9orf117*, *kank1* and *fbp1*) formed a conserved cluster in all species. The analysis of repetitive sequences showed that the number of retroelements and simple repeat per BAC showed its highest value in the subcentromeric region where 53B20, 16E16 and 48K7 BACs were localized. This region contains all the *dmrt* genes, which are associated with sex determination in some species. In addition, the presence of a satellite “chromosome Y” (motif length: 860 bp) was detected in this region. These findings allowed to trace an evolutionary trend for the large metacentric chromosome of *S. senegalensis,* throughout different rearrangements, which could be at an initial phase of differentiation as sex chromosome.

## 1. Introduction

Teleosts are the most diversified group among vertebrates [1], colonizing aquatic environments with a wide range of salinity, temperature or photoperiod conditions and displaying a high variety of adaptations. This variability also reaches sex determination systems, of which a great variety of them have been described in fish that can be (i) simple system with heterogametic male as XX/XY, heterogametic female as ZZ/ZW, and homogametic female and male XX/X0 and ZZ/Z0 respectively, and (ii) multiple system with standard as XX/XY_1_Y_2_ which was observed in neo tropical fish *Hoplias malabaricus* in which XY_1_Y_2_ is a neo-sex chromosome, or constitution or numerical variations (revised in [2]). Pleuronectiformes are a diverse order composed of more than 600 species grouped into 11 families, they have a small genome [3] when compared to other teleost fish, making them an interesting group to study mechanisms of organization and gene expression. Within this order, both ZZ/ZW (e.g., *Cynoglossus semilaevis*, [4] and XX/XY (e.g., *Solea senegalensis*, [5]) sex chromosome determination systems have been described.

Sex determination is a process with many consequences at biological and evolutionary levels, and therefore, a high level of conservation would be expected as it happens with other important biological processes [6]. In fish, specifically, sex determination mechanisms involve, in many cases, more than one gene, as well as environmental factors and epigenetic mechanisms [7].

Sex determination in fish shows considerable variation among species, and six different master genes have described as responsible, *dmy*, *gsdf*, *sox3*, *amhy*, *amhr2* and *sdY* [7,8]. Three of these have been described in different species of *Oryzias*. The first gene described in fish, *dmy* (DM-domain gene on the Y chromosome) has been reported in two species of *Oryzias*, *O. latipes* and *O. curvinotus*, and originated from a duplication of the autosomal gene *dmrt1*. The *gsdf* gene (gonadal soma derived factor on the Y chromosome), was reported in *O. luzonensi* [8]; and *sox3*, which was described on the Y chromosome of *O. dancena*, appeared independently, given that this species belongs to a different lineage [9]. Other genes are *amhy* (Y chromosome-specific anti-müllerian hormone) and *amhr2* (anti-müllerian hormone receptor type II). The former gene is a male-specific duplicate gene that emerged from a duplication event of the autosomal *amh* gene [10]. It is located on a single metacentric/submetacentric chromosome and is expressed in presumptive Sertoli cells of XY individuals of *Odonthestes hatcheri* (Paragonian pejerrey). The *amhr2* gene is expressed in somatic cells surrounding germ cells and it determines sex in *Takifugu rubripes*, likely due to a single-nucleotide difference between the Y and X chromosome that changes an amionacid (His/Asp384) in the kinase domain [11].

The last master sex-determining gene is *sdY* (sexually dimorphic on the Y-chromosome), an immune-related gene identified in the rainbow trout *Oncorhynchus mykiss* [12]. It has also been suggested that *dmrt1* (doublesex and mab-3 related transcription factor 1) could be a sex-determining candidate of in *C. semilaevis*, which has a ZZ/ZW system [4].

The evolution of sex chromosomes has been often been mediated by fusions between sex chromosomes and autosomes of the type Y-A. This type of fusions has been observed at higher rates in fish and squamates reptiles than other sex chromosomes-autosomes fusions, being the XY lineages more frequently fused than ZW [13]. Hence, in two species of fishes of the *Oplegnathus* genus it was observed that multiple X_1_X_2_Y sex chromosomes were formed through a centric fusion of ancestral Y chromosome with an autosome, creating a large neo-Y chromosome [14]. The Neotropical fish *H. malabaricus* (Erythrinidae family) was described as having five karyomorphs, one of these, the (XX/XY_1_Y_2_) multiple systems G, derived by tandem fusion events between the acrocentric proto-sex chromosome pair and the submetacentric autosome pair from ancestral karyotype, [2].

In *S. senegalensis*, which has a XX/XY system, the chromosome 1, has been described as a proto-sex chromosome [15] originated by Robertsonian fusions between two acrocentric chromosomes [16].

It has been postulated that heteromorphic sex chromosomes evolved from an ordinary autosomal pair after acquisition of a sex determination (SD) locus. One of the first events in the evolution of sex chromosomes and what makes the W and Y different from X and Z chromosomes, is the suppression of the meiotic recombination between X and Y as well as Z and W chromosomes due to accumulation of heterochromatin and deletion of heterochromatin [17]. Moreover, other events associated with the evolution of sex chromosomes implied accumulation of TEs and simple repeats around young sex determination locus [17,18,19].

In heteromorphic sex chromosomes the SD system might involve (i) the whole chromosome and be detectable with cytogenetic techniques as described for Neotropical fish [2]; (ii) could be a cryptic region with cytogenetic level as observed in three-spined stickleback [20]; (iii) regions with a few kilobases as reported in *O. latipes* [21] and (iv) be a very tiny differentiation as observed in *T. rubripes* in which the only differentiation between X and Y is the single SNP in the *amhr2* receptor [11].

The karyotype, of *S. senegalensis* has been described as 2*n* = 42 [22], its Fundamental Number (FN) is 60 and is composed by 6 M + 4 SM + 8 ST + 24 T. The largest metacentric chromosome is proposed to have evolved through the Robertsonian fusion of two acrocentric ones [23]. The Zoo-FISH technique (FISH: Fluorescence *In Situ* Hybridization) in two related flatfish species of the Soleidae family, *Dicologlossa cuneata* (2*n* = 50, and FN = 54) and *Dagetichthys lusitanica* (2*n* = 42, and FN = 50) confirmed this prediction [16].

Thus, with this antecedents/precedents, the aim of this work is deepen into the evolution of the metacentric chromosome 1 of *S. senegalensis* through studies of genomic synteny in closely-related flatfish species, *Scophthalmus maximus* and *C. semilaevis*, and also distant/faraway species not belonging to the Pleuronectiformes order (*Tetraodon nigroviridis, Gasterosteus aculeatus, Xiphophorus maculatus, Oryzias latipes, Danio rerio and Lepisosteus oculatus*).

## 2. Results

### 2.1. Obtention of the Integrated Map of Large Metacentric Chromosome 1 of S. senegalensis

**Figure 1:**
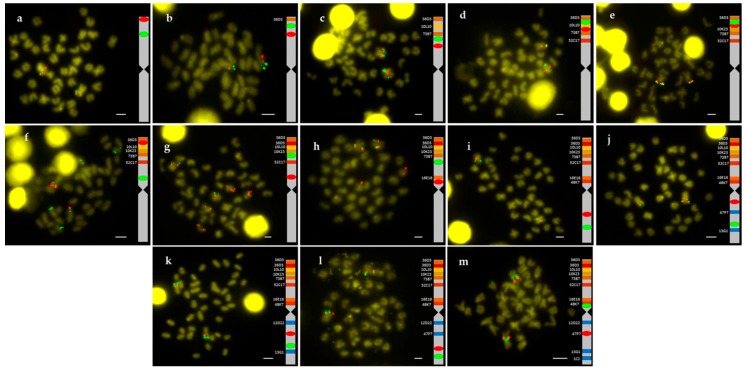
Double FISH-BAC of (**a**) 73B7 (green)/36D3 (red), (**b**) 10L10 (green)/73B7 (red), (**c**) 10K23 (green)/52C17 (red), (**d**) 5K5 (green)/10K23 (red), (**e**) 5K5 (green)/10L10 (red), (**f**) 16E16 (green)/5K5 (red), (**g**) 73B7 (green)/16E16 (red), (**h**) 52C17 (green)/48K7 (red), (**i**) 13G1 (green)/47P7 (red), (**j**) 1C2 (green)/12D22 (red), (**k**) 1C2 (green/47P7 (red), (**l**) 1C2 (green)/13G1 (red), and (**m**) 53B20 (green)/47P7 (red). Bar = 2 µm.

A total of 14 BACs with 88 genes annotated were studied (Table 1). The relative BAC positions on the chromosome were obtained by double FISH-BAC (Figure 1).

All chromosome preparations showed 2*n* = 42. Nine out of the 14 BAC were found in arm 1 (BAC36D3, BAC5K5, BAC10L10, BAC10K23, BAC73B7, BAC52C17, BAC16E16, BAC48K7 and BAC53B20, telomere to centromere direction). The last three clones (BAC16E16, BAC48K7 and BAC53B20) were very close to the centromere and next to them is BAC56H24, located at centromeric position in a previous study [16]. The remaining 4 BAC were located in arm 2: 12D22, 47P7, 13G1 and 1C2 (centromere to telomere direction). The BLAST analysis demonstrated that BAC16E16 overlaps with BAC48K7 and the annotation analysis corroborated that these two BAC clones share the *dmrt2* and *dmrt3* genes. Similarly, the BLAST analysis showed overlap between BAC48K7 and BAC53B20, but not between BAC16E16 and BAC53B20.

**Figure 2:**
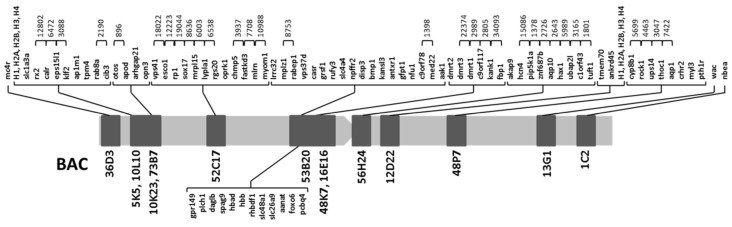
Integrated genetic map of metacentric chromosome 1 of *S. senegalensis*.

To obtain the gene order within each BAC, *C. semilaevis* was used as reference species in the microsynteny study. Due to the high number of BAC located in the arm 1, most genes (67 of 88) belonged to such arm, 9 genes belonged to the centromeric region, and 17 genes were in arm 2 (keeping in mind that the five histone cluster genes were only counted in arm1). Distances between genes were obtained only for genes found in the same contig. Figure 2 shows the integrated genetic map for metacentric chromosome 1.

### 2.2. Synteny Analysis

In order to elucidate the possible evolutionary origin of the metacentric chromosome 1 of *S. senegalensis*, synteny studies were carried out with eight fish species. The integrated map obtained above served as starting point for the synteny analysis. The comparative study showed a high degree of conservation between the genomic regions within the BACs of *S. senegalensis* and genomic regions of close (*C. semilaevis* and *S. maximus*) and more distant species *S. senegalensis* genomic regions (*T. nigroviridis, G. aculeatus, X. maculatus* and *O. latipes*) (Figures S1–S8).

This high conservation was not so evident in *D. rerio* and *L. oculatus.* Eighteen of the 30 genes annotated in BAC53B20 were included in the synteny figures. The other 12 genes were scattered among different chromosomes (Table 2). The distribution of all *S. senegalensis* genes across the chromosomes of the other species is shown in Tables S1–S8.

**Figure 3:**
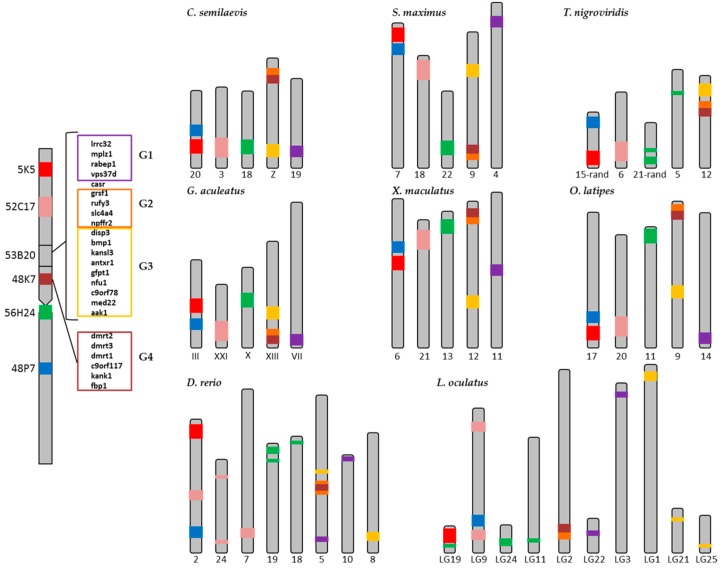
Organization of the BACs and specific regions of the metacentric chromosome 1 of *S. senegalensis* in the species.

One of the most important findings was that six BACs (BAC5K5, BAC52C17, BAC53B20, BAC84K7, BAC56H24, and BAC48P7) were distributed among at least 5 different chromosomes in the reference species (Figure 3), thus allowing us to trace an evolutionary trend for the appearance of the large metacentric chromosome of *S. senegalensis,* throughout different rearrangement events.

BAC5K5 and BAC48P7 BACs, which are located in different arms in *S. senegalensis,* were found near each other in the same chromosome in all species except in *T. nigroviridis* and *D. rerio*. In these two species, BAC5K5 and BAC48P7 were located far away from each other near the two telomeric regions. Moreover, in *L. oculatus,* these two BACs were found in different chromosomes linked to genes representative of other BACs (Figure 3). All genes found within each BAC were conserved among the studied species.

The gene organization of BAC52C17 was conserved among all species, except in *D. rerio* and *L. oculatus*. Genes in this BAC were distributed among three chromosomes in *D. rerio* and in one linkage group in *L. oculatus*, but scattered and located in the extremes. The same situation was observed for BAC56H24, except for *D. rerio*, *L. oculatus* and *T. nigroviridis*.

The most interesting finding in the synteny analysis was obtained in the BAC53B20 and BAC48K7. In all species a group of four genes of BAC53B20 (*grsf1*, *rufy3*, *slc4a4* and *npffr2*) (henceforth/from now on named G2) and the genes of BAC48K7 (*dmrt2*, *dmrt3*, *dmrt1*, *c9orf117*, *kank1* and *fbp1*) (henceforth/from now on named G4) are present in the same cluster. However, in *S. senegalensis*, between G2 and G4 clusters, a group of genes (*disp3*, *bmp1*, *kansl3*, *antxr1*, *gfpt1*, *nfu1*, *c9orf78*, *med22* and *aak1*; from now on named as G3) was observed. These genes are positioned in the same chromosome in six species, but in a different position to the G2-G4 cluster.

The exceptions were *D. rerio*, in which only two genes of G3 (*c9orf78* and *med22*) were in the same chromosome (Chr. 5), and *L. oculatus*, in which all G3 genes were in three different linkage groups (LG1, LG21 and LG25). The position of G2 and G4 was inverted (taking as reference G3) for *T. nigroviridis*, *G. aculeatus* and *X. maculatus* with respect to *C. semilaevis*, *S. maximus* and *O. latipes*. Other genes of BAC53B20 (*lrrc32*, *mplz1*, *rabep1* and *vps37d*) (from now on named G1) were conserved and positioned in different chromosomes in five species, *C. semilaevis*, *S. maximus*, *G. aculeatus*, *X maculatus* and *O. latipes.* In *D. rerio* and *L. oculatus*, these genes were placed in two different chromosomes. For *T. nigroviridis* it was impossible to find the G1 orthologous genes. The remaining genes of BAC53B20 (Table 2) were also located in other different chromosomes.

### 2.3. Repetitive Sequences

**Figure 4:**
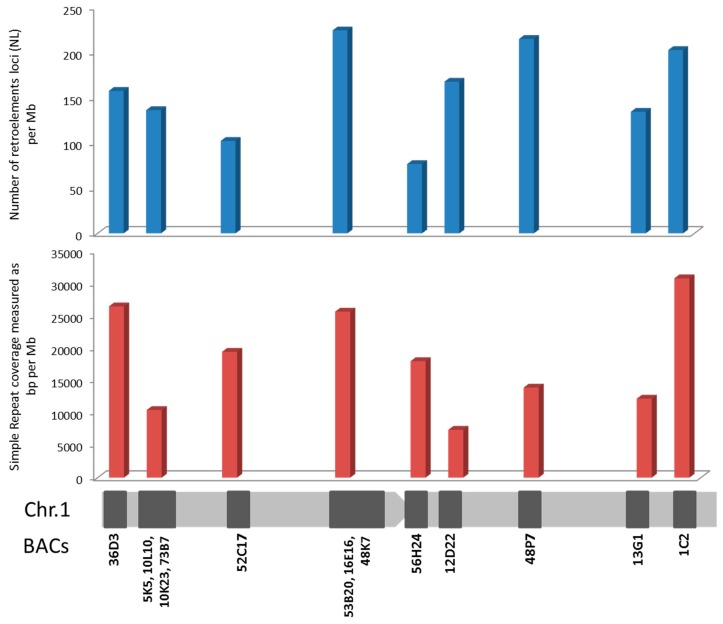
Repetitive elements distribution along *S. senegalensis* chromosome 1.

The 14 BACs listed in Table 1 represent a total sequence length of 2.79 Mb. The number of transposable elements (TEs) and small non-coding RNAs (sRNA elements) was 1297, showing 464.4 loci per BAC and Mb. The number of satellite DNA and low complexity loci was 1449, with an average of 518.8 loci per Mb. The length of these sequences was 58 kb, representing 2.1% of the genome analyzed (Tables S9 and S10). In relation to the repetitive elements distribution along the chromosomes, and taking into account that some BACs overlapped both in FISH and bioinformatics analysis, BACs 5K5, 10L10, 10K23, 73B7 on one hand, and 53B20, 16E16, 48K7 on the other, were grouped for analysis (Figure 4, Table S11). The distribution of DNA transposons, sRNA, satellites and low complexity sequences was homogeneous along the chromosomes and the differences among then were not related to their location in the arms (Figure S9, Table S11). However, the number of loci of retroelements per BAC and Mb (Figure 4) showed the highest value in the subcentromeric region where 53B20, 16E16 and 48K7 BACs were localized (224.34 loci/Mb). This subcentromeric region contains all the *dmrt* genes associated with sex determining in some species. On the other side, the coverage of simple repeats showed three regions with higher values than the rest of the chromosome: telomeric BACs 36D3 and 1C2 (26460.44 and 30811.13 bp/Mb respectively) and again the subcentromeric region containing *dmrt* genes (53B20, 16E16 and 48K7 BACs) (25649.10 bp/Mb) (Figure 4).

It is worth mentioning the presence of a “chromosome Y” satellite (motif length: 860 bp) in the subcentromeric region that contains *dmrt* genes. The BLAST analysis of this satellite sequence showed the existence of this sequence in centromeric position of several vertebrate chromosomes and in the long arm of the human Y chromosome, indicating the presence of this satellite in a sex determining region.

## 3. Discussion

**Figure 5:**
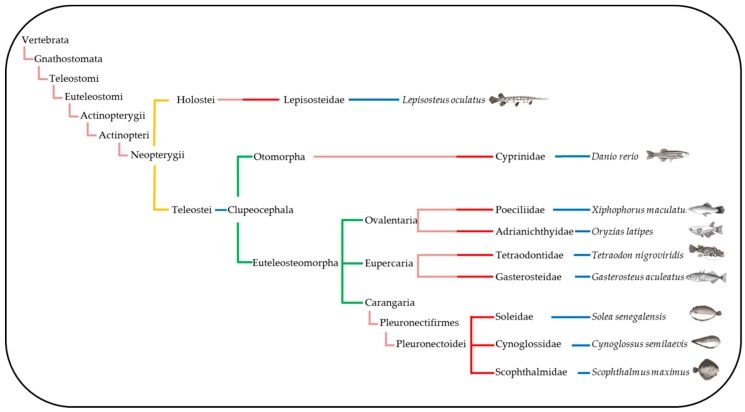
Taxonomic tree showing the species of fishes included in this work.

The comparative genomic study of 14 BAC with 88 genes located in the large metacentric chromosome 1 of *S. senegalensis* was carried out in eight fish species, including the spotted gar, which belongs to a non-teleost fish lineage that did not suffer the teleost genome duplication (TGD) [24]. The taxonomic relation among all species included in the present work is shown in Figure 5. Three out of the nine species were flatfish belonging to the Pleuronectiformes order, thus in the taxonomic tree three families of Pleuronectiformes: Soleidae (*S. senegalensis*), Cynoglossidae (*C. semilaevis*) and Scophthalmidae (*S. maximus*) are/were represented. The Pleuronectiformes have been the subject of controversy regarding the monophyletic or polyphyletic origin of the group. Recently, dates generated by analysis from more than 1000 UCE (Ultraconserved DNA Elements) loci provided strong molecular support for the monophyletic origin of flatfishes and for the single origin of cranial asymmetry [25,26]. Despite the taxonomic proximity of *S. maximus* and *C. semilaevis*, the analysis of the gene groups carried out in the Figure 3, they were more similar to other distant species that to *S. senegalensis.* García-Angulo et al. [16] showed that the metacentric chromosome 1 of *S. senegalensis* hybridized to two acrocentric chromosome pairs of two species of the Soleidae family, thus demonstrating the origin of the metacentric chromosome 1 by a Robertsonian fusion. In addition, more than 80% of the metacentric chromosome 1 genes are distributed in four chromosomes of both *C. semilaevis* and *S. maximus*. Moreover, the conservation of groups of genes in all species studied, which are also conserved in *L. oculatus*, demonstrates a common origin in the evolution of Teleostei and Holostei [27]. Although two acrocentric pairs of both *Dagetichthys* and *Dicologlossa* show hybridization to each arm of chromosome 1 of *S. senegalensis* [16], there are no data about the distribution of BACs in these acrocentric chromosomes. On the other hand Merlo et al. [23] reported pericentric inversions in the chromosome 1 of *S. senegalensis*, which would support that the acrocentric chromosomes of *Dagetichthys* and *Dicologlossa* do not have the same distribution that the BACs in the arms of the metacentric chromosome 1 of *S. senegalensis* have. If this were the case, more differences will be observed between *S. senegalensis* and *Dagetichthys* and *Dicologlossa,* despite all of them belonging to the Soleidae family (taking into account the distribution of BACs on Figure 3), than between the other families of Pleuronectiformes or even among other Euteleosteomorpha families included in this work. Further FISH studies are necessary to assess the distribution of BACs in other Soleidae species.

In relation to the localization of genes from groups G2-G4, the G4 group (Figure 3) contains the *dmrt1-dmrt2-dmrt3* gene cluster and this cluster was found in the Z sex-chromosome of *C. semilaevis*; only the Z chromosome contains a functional copy of *dmrt1* in this species, and it was proposed as an important sex-determining gene in *C. semilaevis* [4]. Moreover, the same cluster, G4, was positioned in the *dmY* region of medaka (except *dmrt1* and *c9orf117* genes), which was described as sex determination (SD) gene that emerged from a segment duplication of a small autosomal region containing the precursor *dmrt1*, followed by an insertion into the proto-Y chromosome [21]. The same *dmY* gene was described in two different species of medaka, *O. latipes* and *Oryzias curvinotus* [8,21]. These findings suggest that the pericentromeric region of the metacentric chromosome 1 of *S. senegalensis* might be a sex-determining zone, but future studies are necessary.

Sex chromosomes are good model systems for the study of evolutionary processes that occur in the absence of recombination. The non-recombining parts of the Y chromosome are unique genomic regions in which the processes of gene degeneration and accumulation of repetitive elements, including microsatellites, are active [28,29,30,31]. Microsatellites are sometimes co-localized with other genomic repeats, especially TE. In humans, microsatellites are associated with other repetitive DNA, especially non- LTR retrotransposons [32,33]. In plants as *Arabidopsis*, rice, soybean, and wheat, however, microsatellites are preferentially associated to non-repetitive DNA regions, indicating that they reside in regions pre-dating genome expansion [34,35]. In fish as *X. maculatus* it has been found a high content of active TEs and satellite sequences on the SD segments of the X and Y chromosomes, and the distribution of long repeats is different between X and Y chromosomes [18,19]. In *O. latipes*, it has also been observed an accumulation of TEs after the formation of the SD region containing the *dmrt1Y* gene [19]. Moreover, the existence of a conserved genetic cascade of SD common to all vertebrates governed by different lineages or species-specific genes such as *dmrt1* has been hypothesized [36]. In this study, a high number of retrotransposons loci and a high abundance of simple repeats elements were found in a region containing *dmrt* genes in the proto-sexual chromosome 1 of *S. senegalensis*. Besides, in this region, a satellite element related with human Y chromosome was observed too. In *Rumex acetosa* (Y_1_Y_2_X system, 12–13 mya), microsatellites expanded on the relatively young and large Y chromosomes [31] but not on the older and small Y chromosome of human (XY system, 150 mya) [37] or *Marchantia polymorpha* (XY system) [38]. In this way, *R. acetosa* data indicates that microsatellite arrays are targets for TE insertions. It has been suggested that early Y chromosome evolution is accompanied by microsatellite expansion and that expansion ability is an intrinsic property of some microsatellites, and the non-recombining regions of the young Y chromosome provide the opportunity for expansion [39], while the revitalizing process of recombination opposes this expansion on other chromosomes [31]. It has also been reported that the accumulation of DNA repetitive sequences increased the size of X and Y chromosomes in the initial phase of differentiation of sex chromosomes [17]. This model can explain sequence patterns on older Y chromosomes, which is characterized by short microsatellite arrays and higher number of TE and it has been proposed that microsatellites served as targets for TE insertions [31]. Support for this is provided by the colocalization of microsatellites with transposable elements in a partial human genome sequence [32].

In this study, simple repeats elements and retroelements were observed in a pericentromeric location in the chromosome 1 of *S. senegalensis* where *dmrt* genes, usually associated to sex determining, are present. This could indicate a non- (or low-rate) recombining region in the genome of *S. senegalensis*, and the accumulation of these repetitive sequences could account for the evolution of the putative sex-determining chromosome of this species.

The synteny data and repetitive sequences analysis could give insights about the evolution of this chromosome which is proposed as neo sex proto-chromosome. The synteny analysis (Figure 3) and the distribution of BACs 5K5, 52C17, 53B20, 48K7, 56H24 and 48P7 in five chromosomes seems to indicate that this distribution is conserved from the ancestral Eutelosteomorpha to the actual teleosts as it was observed in the reference species. This distribution, however, is not present in *S. senegalensis*. It was reported in studies of vertebrate evolution that the genome of the bony vertebrate ancestor contained both macro and micro-chromosomes, some of which remain conserved in *L. oculatus*. These chromosomes suffered fusions in the lineage leading to teleosts after divergence from *L. oculatus*, followed by the TGD of the fused chromosomes and subsequent interchromosomal rearrangements and rediploidization, which led to the formation of macrochromosomes in teleosts, as for example *O. latipes* [27]. These events could lead to establishing the current situation observed in Figure 3, just like for all other species, as included in this work.

**Figure 6:**
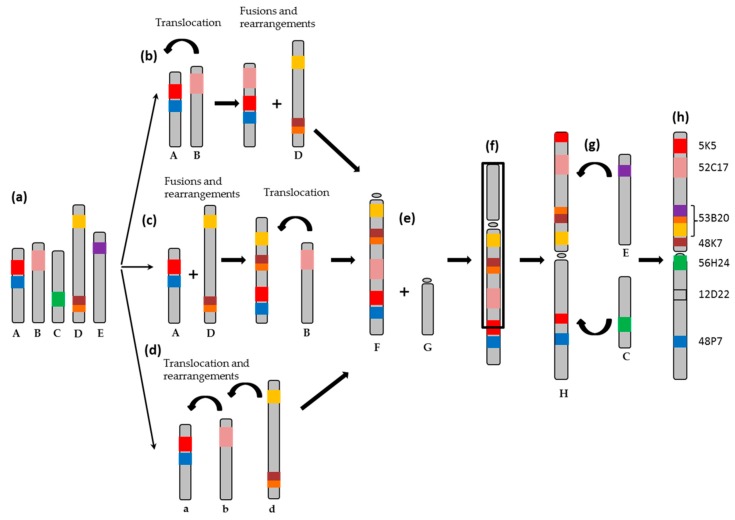
Evolution of the proto sex- chromosome 1 of *S. senegalensis* from ancestral chromosomes after evolution of Teleost fish and Euteleosteomprpha. (**a**) A, B, C, D and E represent ancestral chromosomes. (**b**–**d**) are the proposed paths for the evolution of the chromosome of *S. senegalensis*. (**e**) Robertsonian fusions. (**f**) Pericentric inversions and rearrangements. The black square represents the region in which pericentric inversion occurred. (**g**) Translocation and rearrangements events. (**h**) Current metacentric proto sex-chromosome of *S. senegalensis*.

To explain the evolutionary origin of the proto sex- chromosome 1 of *S. senegalensis* from a common ancestor of Eutelosteomorpha three scenarios (Figure 6a), starting with five ancestral chromosomes, are proposed. The situations described in Figure 6b,c show how translocations, fusions and rearrangements events among “A”, “B”, and “C” chromosomes could have originated a new chromosome called “F”. In the formation of sex chromosomes events such as fusions between sex chromosomes and autosome of the type Y-A has been described [13], or in the formation of (XX/XY_1_Y_2_) multiple system in neo tropical fish [2], or in the X_1_X_2_Y sex system, which was formed through a centric fusion of ancestral Y chromosome with an autosome, creating a large neo-Y chromosome in the *Oplegnathus* genus [14]. In the situation described in Figure 6d only translocations and rearrangements are proposed to originate the “F” chromosome. The next step, as described by Merlo et al. [23], a Robertsonain fusion must have happened between “F” chromosome and another one called “G” (Figure 6e), which is unknown with respect to its gene content, followed by a pericentromeric inversion (Figure 6f). Hence, this type of rearrangement could be related with the evolution of the proto sex chromosome in *S. senegalensis*. For example, a large inversion has been described in the zone corresponding to the sex-determining region of the XY system in the nine-spined stickcleback *Pungitius pungitius* [6], and both, an inversion and a deletion in *G. aculeatus* Y chromosomes, suggesting that the process of sex chromosome evolution can occur rapidly in sex chromosomes evolution and after the acquisition of the sex determining region (SD) [20]. At the same time, translocations and rearrangements with the “C” and also with “E” chromosomes and accumulation of repetitive sequences could have occurred in the pericentromeric region (Figure 6g). This is suggested in our data by the presence of repetitive sequences in the zone of the BAC53B20 and 48K7 (see above). This could lead to the actual proto-sex chromosome of *S. senegalensis* (Figure 6h).

## 4. Materials and Methods

### 4.1. BAC Clones

BACs used in this work were isolated from a BAC library of *S. senegalensis*, composed of 29,184 positive clones distributed in 384-well plates (76 plates in total). The 4D-PCR method [40] was used to isolate BACs. Details of the construction of the BAC library and the strategy used to find and isolate BAC clones are described by García-Cegarra et al. [41]. The name of each BAC indicates the library plate number (first set of digits) and well coordinates (A- and 1- ).

### 4.2. Chromosome Preparation and Double BAC-FISH

Chromosome preparations were carried out using larvae (1–3 days after hatching) of *S. senegalensis.* Larvae were pre-treated with 0.02% colchicine for 3 h to obtain a large number of metaphasic cells. After that, they were treated with KCl (0.4%), and finally they were fixed in a freshly solution of Carnoy (absolute ethanol:acetic acid, 3:1). Prior to hybridization and to isolate the BAC, 50 mL of LB supplemented with chloramphenicol were inoculated from a pre-culture (8 h growth at 37 °C in a shaker). DNA-BAC were purified using Plasmid Midi Kit (Qiagen, Hilden, Germany), following the manufacturer’s instructions. This DNA was labeled by Nick Translation using the DIG or BIO -Nik Translation Mix (Roche Molecular Biochemical) and used as probe as described in the manufacturer’s instructions.

Pre-treatment of chromosome preparations and hybridization was carried out as described by García-Cegarra et al. [41]. Then, 2-colour FISH was used. For digoxigenin-labeled probes, hybridization sites were detected using anti-Digoxigenin/Digoxin (Vector Labs, Burlingame, CA, USA) at 1:200 and anti-Goat IgG (vector Labs) at 1:100. Avidin FITC (Vector Labs) at 1:200 and biotinylated anti- avidin (Vector labs) at 1:100 were used for biotin-labeled probes. These antibodies were prepared in TNFM (4x SSC, 0.05% Tween 20, 5% skim milk). Slides were then washed in TNFM, dehydrated with ethanol (70%, 90% and 100%) and counterstained with 0.4 mg/mL DAPI-Vectashield (ATOM). Images were recorded using a Zeiss PALM MicroBeam microdissector and fluorescence microscopes equipped with an AxioCam MRm digital camera.

### 4.3. Sequencing and Bioinformatic Analysis

The DNA-BAC extracted with the Large Construct kit (Qiagen, Hilden, Germany) was sequenced using the Illumina sequencing platform (Illumina, San Diego, CA, USA). The BAC clones were deposited in the GenBank database under the accession numbers AC278047 to AC278120. The functional and structural annotation of the genes identified in each BAC was carried out as described by García-Angulo et al. [41].

For the analysis of micro-synteny, *C. semilaevis* was selected as the genome reference and the ENSEMBL database was used to obtain the order of the genes. The order of the contigs within each BAC and the distance between the genes within each contig of *S*. *senegalensis* were estimated using the information provided by the Geneious R11 [42]. The “nucleotide BLAST” tool was used to found overlapping among contiguous BACs and the study of the repetitive sequences.

### 4.4. Repetitive Elements Analysis

After determining the chromosome location of BAC clones, using the FISH technique, a homology-based approach using the Repbase database (https://www.girinst.org/repbase/, release 23.07) with RepeatMasker software (http://www.repeatmasker.org/) [43] was applied. Distribution analysis of repetitive elements as TEs (Retroelements and DNA transposons), sRNA, DNA satellite (measured as satellites and simple repeats) and low complexity sequences, in *S. senegalensis* metacentric chromosome, was possible by using both the information of BAC clone position, obtained afterthe double BACFISH technique, and the coordinates of the elements from the RepeatMasker software. The average number of TEs and sRNA was calculated as the total number of identified loci in relation to the BAC sequences length analyzed and normalized by Mb. The coverage of DNA satellite and low complexity elements was calculated as the quantity of sequences (bp) in relation to the BAC sequences length analyzed, and again normalized by Mb.

### 4.5. Synteny Analyses

For comparative genomic analysis eight species of fish were used, *Cynoglossus semilaevis* (2*n* = 42) or smooth tongue sole, *Scophthalmus maximus* (2*n* = 44) or turbot, *Tetraodon nigroviridis* (2*n* = 42) or smooth pufferfish, *Gasterosteus aculeatus* (2*n* = 42) or three-spined stickleback, *Xiphophorus maculatus* (2*n* = 48) or platyfish, *Oryzias latipes* (2*n* = 48) or medaka, *Danio rerio* (2*n* = 50) or zebrafish and *Lepisosteus oculatus* (2*n* = 58, [27]) or spotted gar. The lineages of these references species and *S. senegalensis* were obtained from the National Center for Biotechnology Information (NCBI) database to obtain the taxonomic tree (Figure 5).

The ENSEMBL database and Genome Data Viewer (NCBI) were applied for comparative genomic analysis. The data were used to identify reorganizations within the chromosomes of reference species.

## 5. Conclusions

The evolution of the sex-proto chromosome of *S. senegalensis*, which belong to the Soleidae family, is very different from the evolution of the karyotype of the other families of Pleuronectiformes.

Cytogenetic events, fusions and pericentric inversions reported previously, the analysis synteny carried out (Figure 3) and the detection of simple repeat elements and retroelements in a pericentromeric location in the chromosome 1 of *S. senegalensis*, in the region where *dmrt* genes have been located, indicates that this chromosome is in an initial phase of differentiation as sex chromosome.

## Figures and Tables

**Table 1:** BAC clones located on the metacentric chromosome 1.

Name of BAC	Gene Annotation	References
36D3	*mcr4*	[16]
5K5	*H1, H2a, H2b, H3, H4, slc1a3a, rx2, calr, eps15l1, klf2, ap1m1, tpm4, rab8a, cib3*	[16,23]
10L10	*rx2, calr, eps15l1, klf2*	[16]
10K23	*calr, eps15l1, otos, apod, arhgap21, opn3*	[15]
73B7	*otos, apod, arhgap21*	[16]
52C17	*vps41, esco1, rp1, sox17, mrpl15, lypla1, rgs20, oprk1, chmp5, fastkd3, mlrn, myom1*	[16]
53B20	*lrrc32, mplz1, rabep1, vps37d, casr, gpr149, plch1, grsf1, rufy3, slc4a4, npffr2, daglb, spag9, hbad, hbb, rhbdf1, slc48a1, slc26a9, aanat, foxo6, pcbp4, disp3, bmp1, kansl3, antxr1, gfpt1, nfu1, c9orf78, med22, aak1*	This work
16E16	*dmrt2, dmrt3*	[15,16]
48K7	*dmrt2, dmrt3, dmrt1, c9orf117, kank1, fbp1*	[16]
56H24	*akap9, hcn4, pip5k1a, znf687b, aqp10, hax1, ubap2l, c1orf43, tuft1*	[16]
12D22	*H1, H2a, H2b, H3, H4, tmem70, ankrd45*	[23]
48P7	*cyp8b1, rock1, ups14, thoc1, aqp1, crhr2, myl3, pth1r*	[16]
13G1	*wac*	[16]
1C2	*nbea*	[16]

**Table 2:** Distribution of 12 genes out of 30 annotated in BAC53B20 on the chromosomes of the reference species.

*Genes*	*C. semilaevis*	*S. maximus*	*T. nigroviridis*	*G. aculeatus*	*X. maculatus*	*O. latipes*	*D. rerio*	*L. oculatus*
*gpr149*	*4*	*3*	*16*	*I*	*18*	*13*	*^1^*	*LG14*
*plch1*	*4*	*3*	*16*	*I*	*18*	*13*	*18*	*LG14*
*daglb*	*8*	*18*	*2*	*V*	*10*	*19*	*12*	*^1^*
*spag9*	*8*	*18*	*2*	*V*	*10*	*19*	*12*	*LG10*
*hbad*	*8*	*18*	*3*	*XI*	*10*	*8*	*3*	*LG13*
*hbb*	*8*	*18*	*3*	*XI*	*10*	*19*	*3*	*LG13*
*rhbdf1*	*17*	*18*	*3*	*XI*	*10*	*8*	*3*	*LG13*
*slc48a1*	*10*	*11*	*11*	*XII*	*1*	*7*	*23*	*LG4*
*slc26a9*	*^1^*	*11*	*9*	*XII*	*1*	*7*	*23*	*LG3*
*aanat*	*9*	*8*	*3*	*XI*	*16*	*8*	*3*	*LG13*
*foxo6*	*13*	*7*	*^1^*	*X*	*13*	*11*	*19*	*LG6*
*pcbp4*	*^1^*	*6*	*9*	*XII*	*1*	*7*	*22*	*LG5*

*^1^* This gene was not found.

## Supplementary Materials

Supplementary materials can be found at https://www.mdpi.com/1422-0067/20/20/5111/s1.
