# Supplementary material for: Evolution of the Proto Sex-Chromosome in Solea senegalensis
Source: Int J Mol Sci. 2019 Oct 15;20(20):5111. doi: 10.3390/ijms20205111 (PMC6829477; doi:10.3390/ijms20205111)
Supplement: Supplementary file 1 [file ijms-20-05111-s001.zip › ijms-615179-final sup/ijms-615179-final sup legend.docx]

**Supplementary Materials Legends**

**Figure S1**. Syntenic map of the metacentric chromosome 1 of *S. senegalensis* respect to *C. Semilaevis*. Between parenthesis are shown the % of the genes obtained from Table S1.

**Figure S2**. Syntenic map of the metacentric chromosome 1 of *S. senegalensis* respect to *S. maximus*. Between parenthesis are shown the % of the genes obtained from Table S2.

**Figure S3**. Syntenic map of the metacentric chromosome 1 of *S. senegalensis* respect to *T. nigroviridis*. Between parenthesis are shown the % of the genes obtained from Table S3.

**Figure S4**. Syntenic map of the metacentric chromosome 1 of *S. senegalensis* respect to *G. aculeatus*. Between parenthesis are shown the % of the genes obtained from Table S4.

**Figure S5**. Syntenic map of the metacentric chromosome 1 of *S. senegalensis* respect to *X. maculatus*. Between parenthesis are shown the % of the genes obtained from Table S5.

**Figure S6**. Syntenic map of the metacentric chromosome 1 of *S. senegalensis* respect to *O. latipes*. Between parenthesis are shown the % of the genes obtained from Table S6.

**Figure S7**. Syntenic map of the metacentric chromosome 1 of *S. senegalensis* respect to *D. rerio*. Between parenthesis are shown the % of the genes obtained from Table S7.

**Figure S8**. Syntenic map of the metacentric chromosome 1 of *S. senegalensis* respect to *L. oculatus*. Between parenthesis are shown the % of the genes obtained from Table S8.

**Figure S9.** Repetitive elements distribution along *S. senegalensis* chromosome 1. (**a**) Number of DNA transposon loci (NL) per Mb. (**b**) Number of sRNA loci (NL) per Mb. (**c**) Satellite coverage per Mb. (**d**) Low complexity coverage per Mb.

**Table S1**. Distribution (%) of genes in each BAC of *S. senegalensis* found in each chromosome of *C. semilaevis*.

**Table S2**. Distribution (%) of genes in each BAC of *S. senegalensis* found in each chromosome of *S. maximus.*

**Table S3**. Distribution (%) of genes in each BAC of *S. senegalensis* found in each chromosome of *T. nigroviridis.*

**Table S4**. Distribution (%) of genes in each BAC of *S. senegalensis* found in each chromosome of *G. aculeatus*

**Table S5**. Distribution (%) of genes in each BAC of *S. senegalensis* found in each chromosome of *X. maculatus*.

**Table S6**. Distribution (%) of genes in each BAC of *S. senegalensis* found in each chromosome of *O. latipes*.

**Table S7**. Distribution (%) of genes in each BAC of *S. senegalensis* found in each chromosome of *D. rerio*.

**Table S8.** Distribution (%) of genes in each BAC of *S. senegalensis* found in each chromosome of *L. oculatus*.

**Table S9**. Repetitive elements distribution along *S. senegalensis* chromosome 1. (**a**) Number of DNA transposon loci (NL) per Mb. (**b**) Number of sRNA loci (NL) per Mb. (**c**) Satellite coverage per Mb. (**d**) Low complexity coverage per Mb.

**Table S10**. Satellite, Simple Repeats and Low Complexity coverage normalized by Mb of BAC sequences.

**Table S11**. Analysis of repetitive elements by pooling overlapping BACs (5K5, 10L10, 10K23, 73B7 and 53B20, 16E16, 48K7 ) showing number of loci per Mb of Retroelementss, DNA transposons, Small RNA and coverage (bp per Mb) of Satellites, Simple Repeats and Low Complexity elements in the chromosome 1 of *S. senegalensis*.
