# Supplementary material for: Evolution of the Proto Sex-Chromosome in Solea senegalensis
Source: Int J Mol Sci. 2019 Oct 15;20(20):5111. doi: 10.3390/ijms20205111 (PMC6829477; doi:10.3390/ijms20205111)
Supplement: Supplementary file 1 [file ijms-20-05111-s001.zip › ijms-615179-final sup/Table S10.docx]

**Table S10**. Satellite, Simple Repeats and Low Complexity coverage normalized by Mb of BAC sequences.

|  |  | **Coverage** | | | | | |
| --- | --- | --- | --- | --- | --- | --- | --- |
| **BAC** | **Length** | **Satellites (bp)** | **Satellites (bp/Mb)** | **Simple repeats (SSR) (bp)** | **Simple repeats (SSR) (bp/Mb)** | **Low complexity (bp)** | **Low complexity (bp/Mb)** |
| 36D3 | 63491 | 0 | 0 | 1680 | 26460,44321 | 594 | 9355,656707 |
| 5K5 | 582745 | 16 | 27,45626303 | 5045 | 8657,302937 | 396 | 679,54251 |
| 10L10 | 65053 | 0 | 0 | 1229 | 18892,28783 | 71 | 1091,417767 |
| 10K23 | 114984 | 123 | 1069,714047 | 1218 | 10592,77813 | 154 | 1339,316775 |
| 73B7 | 74590 | 201 | 2694,731197 | 1228 | 16463,33289 | 202 | 2708,13782 |
| 52C17 | 185957 | 66 | 354,9207613 | 3612 | 19423,8453 | 231 | 1242,222664 |
| 53B20 | 966994 | 4447 | 4598,787583 | 24694 | 25536,86993 | 2357 | 2437,450491 |
| 16E16 | 43095 | 0 | 0 | 1595 | 37011,25421 | 117 | 2714,932127 |
| 48K7 | 180050 | 138 | 766,4537628 | 4237 | 23532,35212 | 518 | 2876,978617 |
| 56H24 | 156535 | 2161 | 13805,21928 | 2814 | 17976,8103 | 310 | 1980,387773 |
| 12D22 | 53688 | 0 | 0 | 395 | 7357,323797 | 0 | 0 |
| 48P7 | 246624 | 0 | 0 | 3424 | 13883,48255 | 302 | 1224,536136 |
| 13G1 | 14876 | 69 | 4638,343641 | 181 | 12167,24926 | 27 | 1815,004033 |
| 1C2 | 44432 | 217 | 4883,867483 | 1369 | 30811,12712 | 51 | 1147,82139 |
| TOTAL | 2793114 | 7438 |  | 52721 |  | 5330 |  |
| Grouped |  | 58051 | | | | | |
| pb/Mb |  | 0,020783613 | | | | | |
