# Supplementary material for: Evolution of the Proto Sex-Chromosome in Solea senegalensis
Source: Int J Mol Sci. 2019 Oct 15;20(20):5111. doi: 10.3390/ijms20205111 (PMC6829477; doi:10.3390/ijms20205111)
Supplement: Supplementary file 1 [file ijms-20-05111-s001.zip › ijms-615179-final sup/Table S11.docx]

**Table S11**. Analysis of repetitive elements by pooling overlapping BACs (5K5, 10L10, 10K23, 73B7 and 53B20, 16E16, 48K7 ) showing number of loci per Mb of Retroelementss, DNA transposons, Small RNA and coverage (bp per Mb) of Satellites, Simple Repeats and Low Complexity elements in the chromosome 1 of *S. senegalensis*.

|  |  | **NL per Mb** | | | **Coverage (pb per Mb)** | | |
| --- | --- | --- | --- | --- | --- | --- | --- |
| **BAC** | **Length** | **Retroelements** | **DNA transposons** | **Small RNA** | **Satellites** | **Simple repeats (SSR)** | **Low complexity** |
| 36D3 | 63491 | 157,50 | 551,26 | 0,00 | 0,00 | 26460,44 | 9355,66 |
| 5K5, 10L10, 10K23, 73B7 | 837372 | 136,14 | 173,16 | 20,30 | 406,03 | 10413,53 | 982,84 |
| 52C17 | 185957 | 102,17 | 311,90 | 0,00 | 354,92 | 19423,85 | 1242,22 |
| 53B20, 16E16, 48K7 | 1190139 | 224,34 | 347,86 | 5,88 | 3852,49 | 25649,10 | 2513,99 |
| 56H24 | 156535 | 76,66 | 223,59 | 0,00 | 13805,22 | 17976,81 | 1980,39 |
| 12D22 | 53688 | 167,64 | 223,51 | 0,00 | 0,00 | 7357,32 | 0,00 |
| 48P7 | 246624 | 214,90 | 259,50 | 0,00 | 0,00 | 13883,48 | 1224,54 |
| 13G1 | 14876 | 134,44 | 201,67 | 0,00 | 4638,34 | 12167,25 | 1815,00 |
| 1C2 | 44432 | 202,56 | 270,08 | 0,00 | 4883,87 | 30811,13 | 1147,82 |
