# Supplementary material for: Evolution of the Proto Sex-Chromosome in Solea senegalensis
Source: Int J Mol Sci. 2019 Oct 15;20(20):5111. doi: 10.3390/ijms20205111 (PMC6829477; doi:10.3390/ijms20205111)
Supplement: Supplementary file 1 [file ijms-20-05111-s001.zip › ijms-615179-final sup/Table S5.docx]

**Table S5**. Distribution (%) of genes in each BAC of *S. senegalensis* found in each chromosome of *X. maculatus*.

| ***Solea senegalensis*** | | ***Xiphophorus maculatus*** | | |
| --- | --- | --- | --- | --- |
| Total number of genes: 88 | | Total number of genes in *X. maculatus*: 88 (date used to calculate the %) | | |
| **BAC** | **genes** | **nº genes** | **%** | **Chr. *X. maculatus*** |
| 36D3 | *mc4r* | 1 | 1 | 21 |
| 5K5, 10L10, 10K23 | *H1, H2A, H3, H4, H1* | 5 | 6 | 14 |
|  | *slc1a3a* | 9 | 10 | 6 |
|  | *rx2* |  |  |  |
|  | *calr* |  |  |  |
|  | *eps15l1* |  |  |  |
|  | *klf2* |  |  |  |
|  | *ap1m1* |  |  |  |
|  | *tpm4* |  |  |  |
|  | *rab8a* |  |  |  |
|  | *cib3* |  |  |  |
| 10K23, 73B7 | *otos* | 4 | 5 | 21 |
|  | *apod* |  |  |  |
|  | *arhgap21* |  |  |  |
|  | *opn3* |  |  |  |
| 52C17 | *vps41* | 12 | 14 | 21 |
|  | *esco1* |  |  |  |
|  | *rp1* |  |  |  |
|  | *sox17* |  |  |  |
|  | *mrpl15* |  |  |  |
|  | *lypla1* |  |  |  |
|  | *rgas20* |  |  |  |
|  | *oprk1* |  |  |  |
|  | *chmp5* |  |  |  |
|  | *fastkd3* |  |  |  |
|  | *mlrn* |  |  |  |
|  | *myom1* |  |  |  |
| 53B20 | *lrrc32* | 5 | 6 | 11 |
|  | *mplz1* |  |  |  |
|  | *rabep1* |  |  |  |
|  | *vps37d* |  |  |  |
|  | *casr* |  |  |  |
|  | *grsf1* | 13 | 15 | 12 |
|  | *rufy3* |  |  |  |
|  | *slc4a4* |  |  |  |
|  | *npffr2* |  |  |  |
|  | *disp3* |  |  |  |
|  | *bmp1* |  |  |  |
|  | *kansl3* |  |  |  |
|  | *antxr1* |  |  |  |
|  | *gfpt1* |  |  |  |
|  | *nfu1* |  |  |  |
|  | *c9orf78* |  |  |  |
|  | *med22* |  |  |  |
|  | *aak1* |  |  |  |
|  | *gpr149* | 2 | 2 | 18 |
|  | *plch1* |  |  |  |
|  | *daglb* | 5 | 6 | 10 |
|  | *spag9* |  |  |  |
|  | *hbad* |  |  |  |
|  | *hbb* |  |  |  |
|  | *rhbdf1* |  |  |  |
|  | *slc48a1* | 3 | 3 | 1 |
|  | *slc26a9* |  |  |  |
|  | *pcbp4* |  |  |  |
|  | *aanat* | 1 | 1 | 16 |
|  | *foxo6* | 1 | 1 | 13 |
| 16E16, 48K7 | *dmrt2* | 6 | 7 | 12 |
|  | *dmrt3* |  |  |  |
|  | *dmrt1* |  |  |  |
|  | *c9orf117* |  |  |  |
|  | *kank1* |  |  |  |
|  | *fbp1* |  |  |  |
| 56H24 | *akap9* | 9 | 10 | 13 |
|  | *hcn4* |  |  |  |
|  | *pip5k1a* |  |  |  |
|  | *znf687b* |  |  |  |
|  | *aqp10* |  |  |  |
|  | *hax1* |  |  |  |
|  | *ubap2l* |  |  |  |
|  | *c1orf43* |  |  |  |
|  | *tuft1* |  |  |  |
| 12D22 | *(H1, H2A, H3, H4, H1)** | 5 | 6 | 14 |
|  | *tmem70* | 2 | 2 | 6 |
|  | *ankrd45* |  |  |  |
| 48P7 | *cyp8b1* | 8 | 9 | 6 |
|  | *rock1* |  |  |  |
|  | *ups14* |  |  |  |
|  | *thoc1* |  |  |  |
|  | *aqp1* |  |  |  |
|  | *crhr2* |  |  |  |
|  | *myl3* |  |  |  |
|  | *pth1r* |  |  |  |
| 13G1 | *wac* | 1 | 1 | 21 |
| 1C2 | *nbea* | 1 | 1 | 18 |
