# Supplementary material for: Evolution of the Proto Sex-Chromosome in Solea senegalensis
Source: Int J Mol Sci. 2019 Oct 15;20(20):5111. doi: 10.3390/ijms20205111 (PMC6829477; doi:10.3390/ijms20205111)
Supplement: Supplementary file 1 [file ijms-20-05111-s001.zip › ijms-615179-final sup/Table S9 .docx]

**Table S9**. Repetitive elements distribution along *S. senegalensis* chromosome 1. (**a**) Number of DNA transposon loci (NL) per Mb. (**b**) Number of sRNA loci (NL) per Mb. (**c**) Satellite coverage per Mb. (**d**) Low complexity coverage per Mb.

|  |  | **Number of loci (NL)** | | | | | |
| --- | --- | --- | --- | --- | --- | --- | --- |
| **BAC** | **Length** | **Retroelements** | **DNA transposons** | **Small RNA** | **Satellites** | **Simple repeats** | **Low complexity** |
| 36D3 | 63491 | 10 | 35 | 0 | 0 | 37 | 7 |
| 5K5 | 582745 | 60 | 70 | 13 | 1 | 133 | 9 |
| 10L10 | 65053 | 19 | 19 | 0 | 0 | 34 | 2 |
| 10K23 | 114984 | 28 | 26 | 1 | 2 | 36 | 4 |
| 73B7 | 74590 | 7 | 30 | 3 | 3 | 35 | 5 |
| 52C17 | 185957 | 19 | 58 | 0 | 1 | 84 | 5 |
| 53B20 | 966994 | 230 | 368 | 5 | 16 | 573 | 51 |
| 16E16 | 43095 | 11 | 8 | 0 | 0 | 42 | 3 |
| 48K7 | 180050 | 26 | 38 | 2 | 1 | 119 | 14 |
| 56H24 | 156535 | 12 | 35 | 0 | 10 | 66 | 7 |
| 12D22 | 53688 | 9 | 12 | 0 | 0 | 12 | 0 |
| 48P7 | 246624 | 53 | 64 | 0 | 0 | 91 | 9 |
| 13G1 | 14876 | 2 | 3 | 0 | 1 | 6 | 1 |
| 1C2 | 44432 | 9 | 12 | 0 | 2 | 26 | 1 |
| TOTAL | 2793114 | 495 | 778 | 24 | 37 | 1294 | 118 |
| Grouped |  | 1297 | | | 1449 | | |
| NL / Mb |  | 464,36 | | | 518,78 | | |
