# Supplementary figures and images for: Evolution of the Proto Sex-Chromosome in Solea senegalensis
Source: Int J Mol Sci. 2019 Oct 15;20(20):5111. doi: 10.3390/ijms20205111 (PMC6829477; doi:10.3390/ijms20205111)

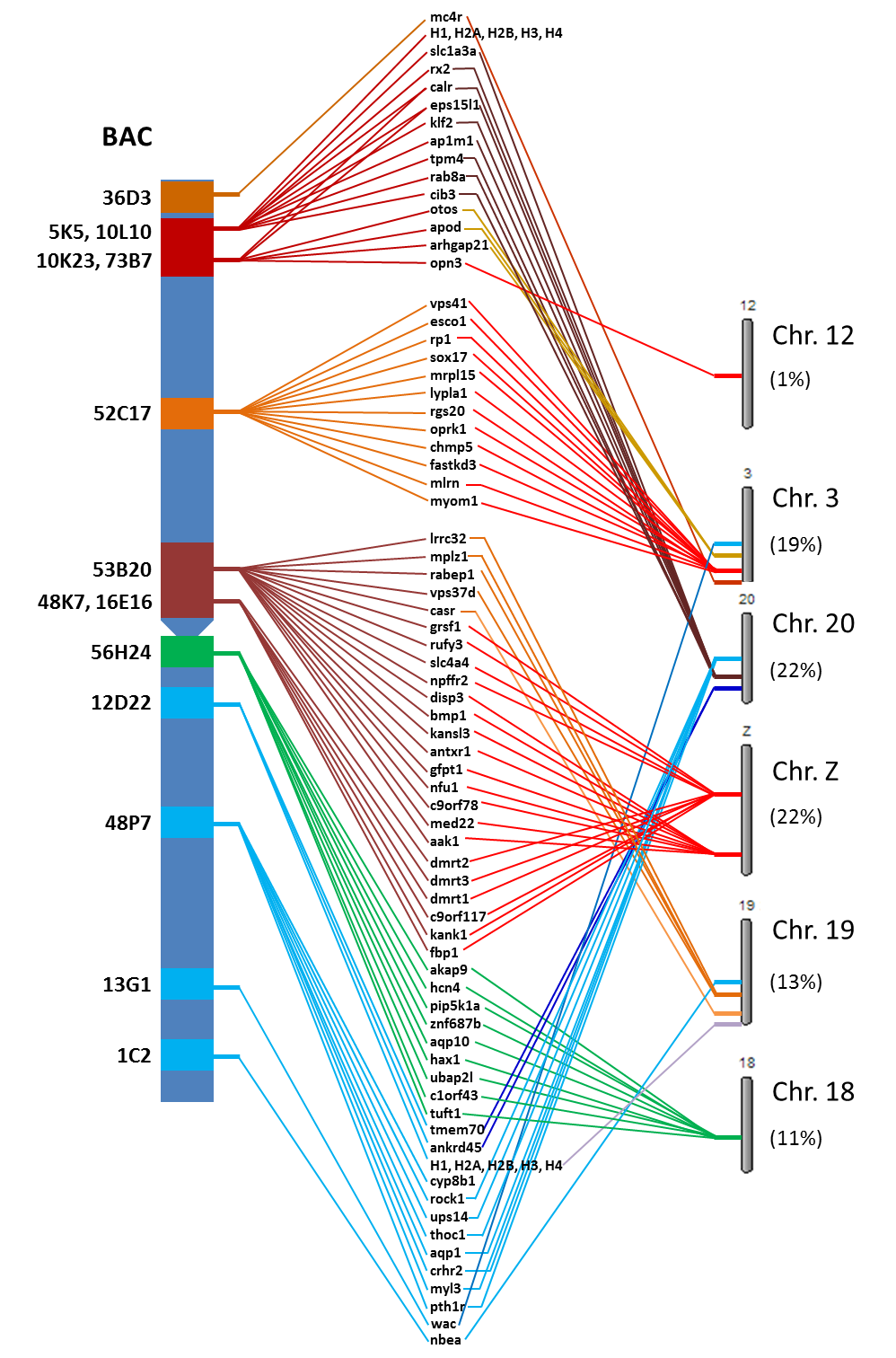

Supplement: Supplementary file 1 [file ijms-20-05111-s001.zip › ijms-615179-final sup/Figure S1.png]

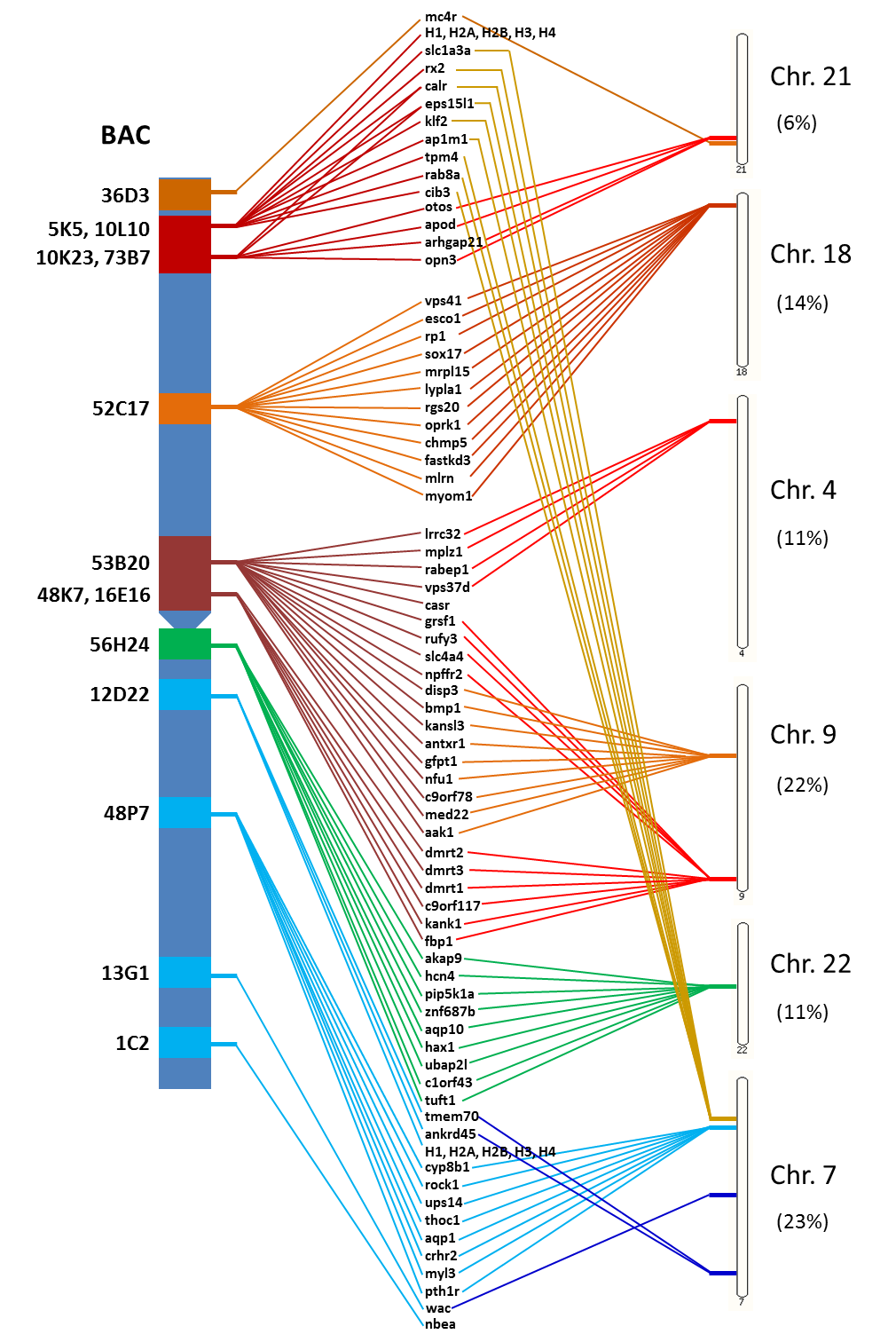

Supplement: Supplementary file 1 [file ijms-20-05111-s001.zip › ijms-615179-final sup/Figure S2.png]

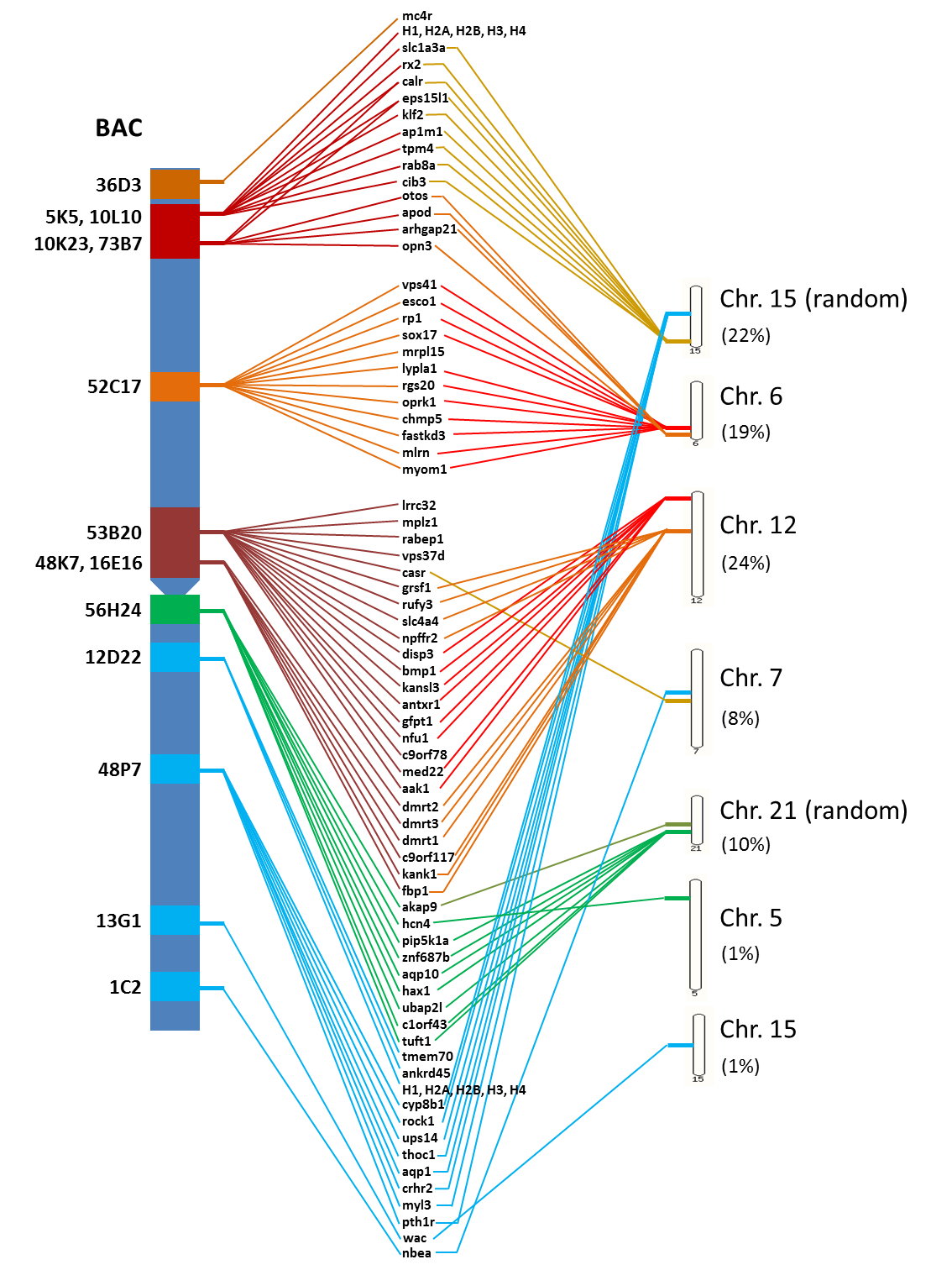

Supplement: Supplementary file 1 [file ijms-20-05111-s001.zip › ijms-615179-final sup/Figure S3.png]

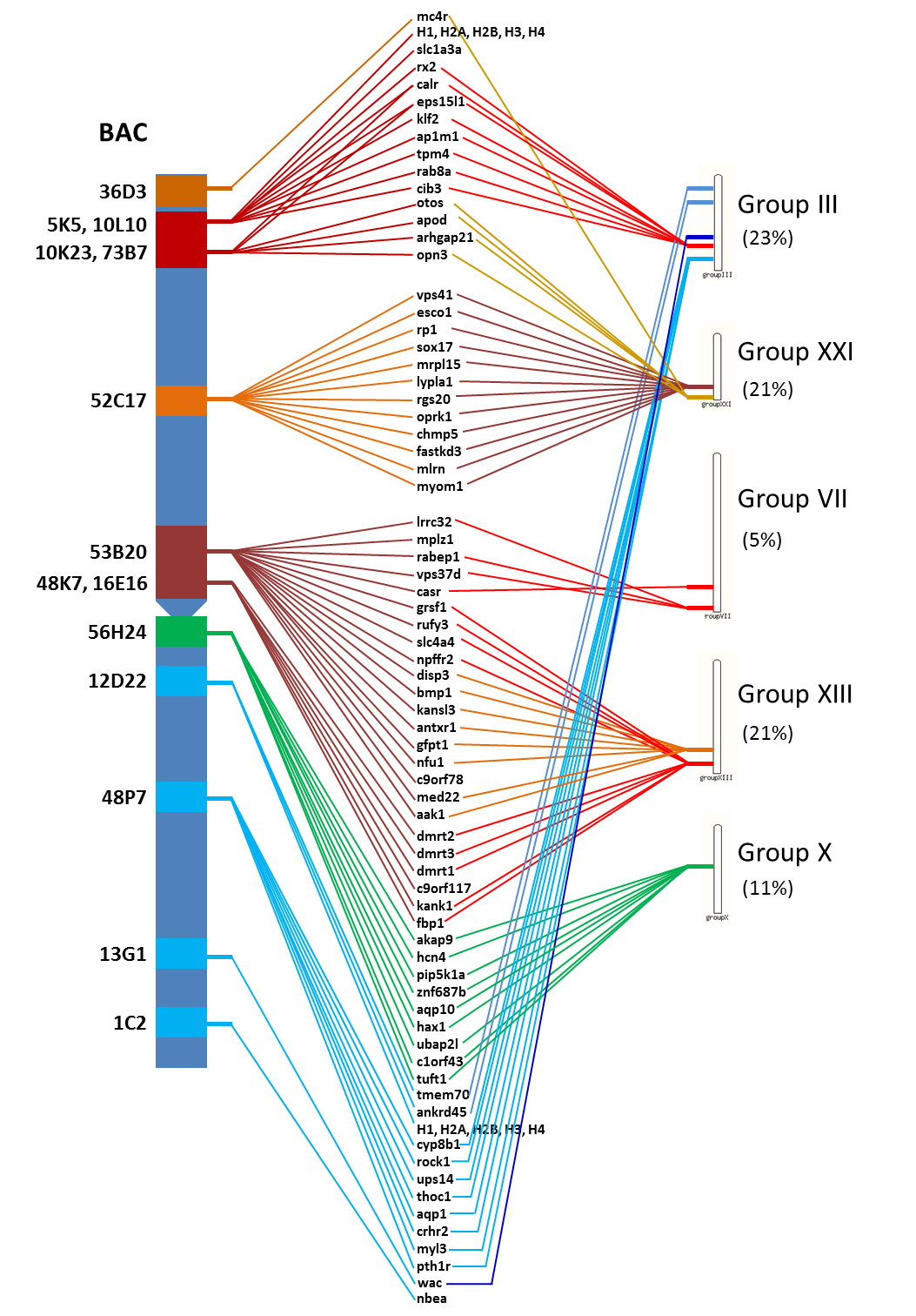

Supplement: Supplementary file 1 [file ijms-20-05111-s001.zip › ijms-615179-final sup/Figure S4.png]

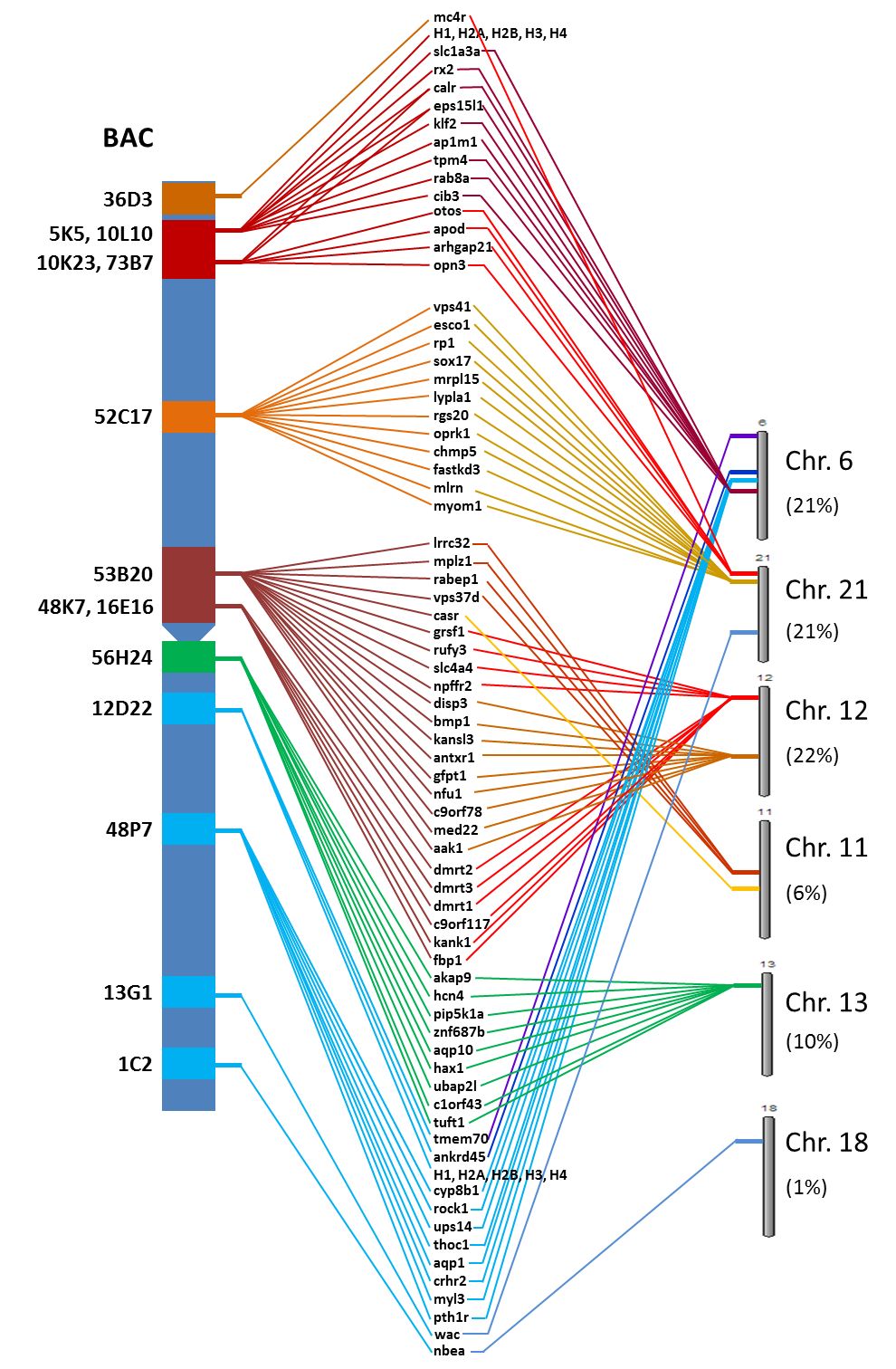

Supplement: Supplementary file 1 [file ijms-20-05111-s001.zip › ijms-615179-final sup/Figure S5.png]

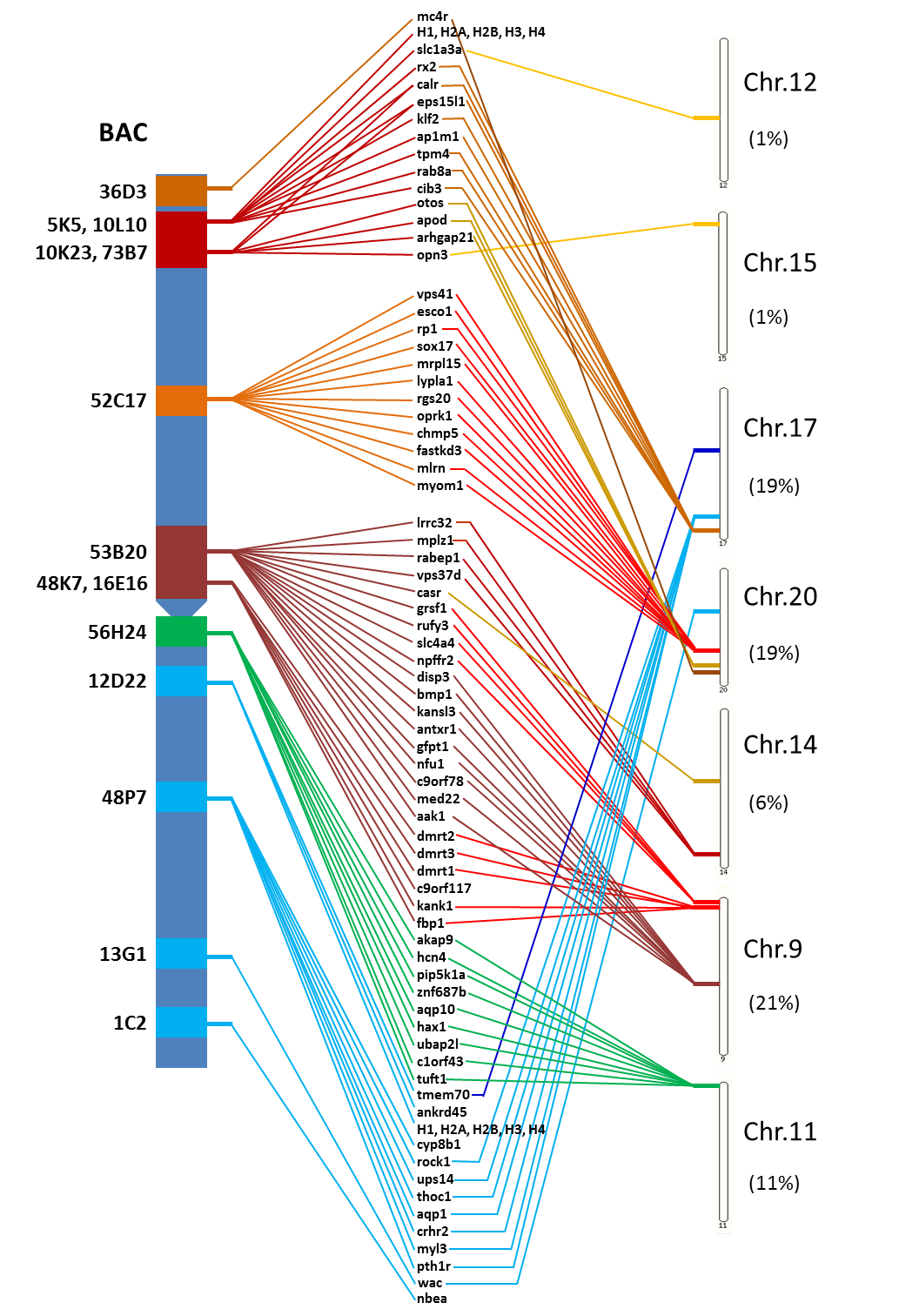

Supplement: Supplementary file 1 [file ijms-20-05111-s001.zip › ijms-615179-final sup/Figure S6.png]

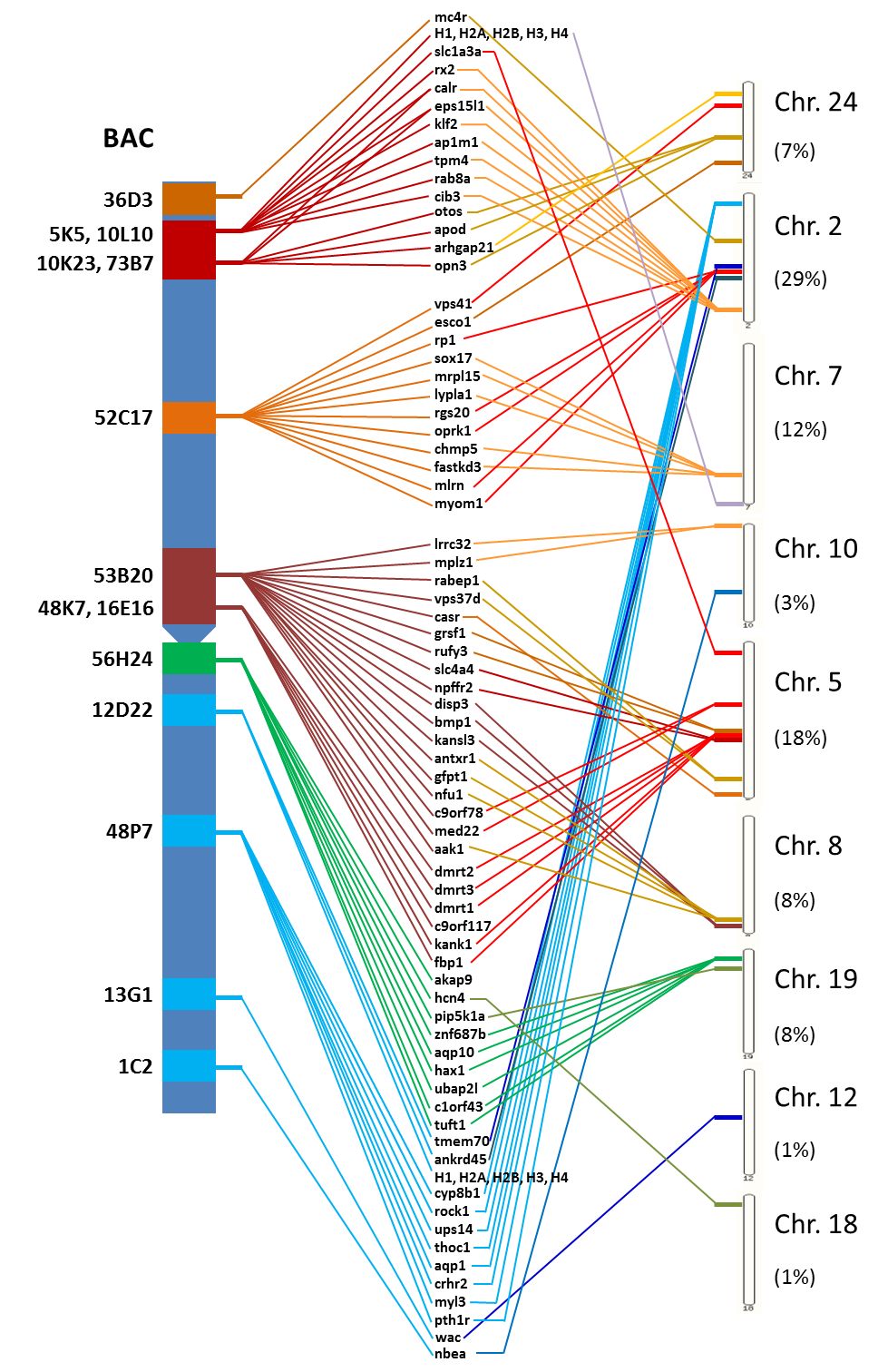

Supplement: Supplementary file 1 [file ijms-20-05111-s001.zip › ijms-615179-final sup/Figure S7.png]

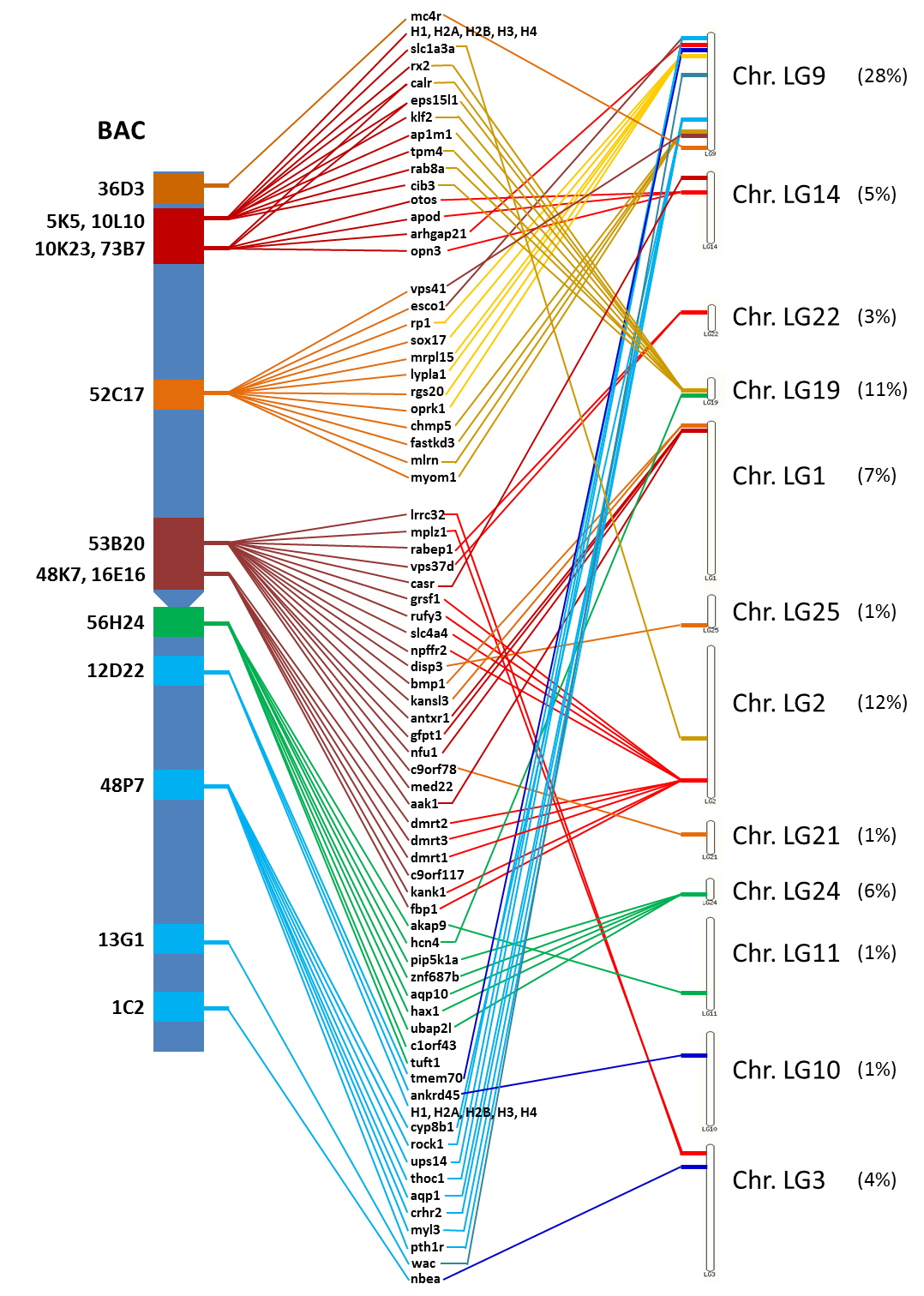

Supplement: Supplementary file 1 [file ijms-20-05111-s001.zip › ijms-615179-final sup/Figure S8.png]

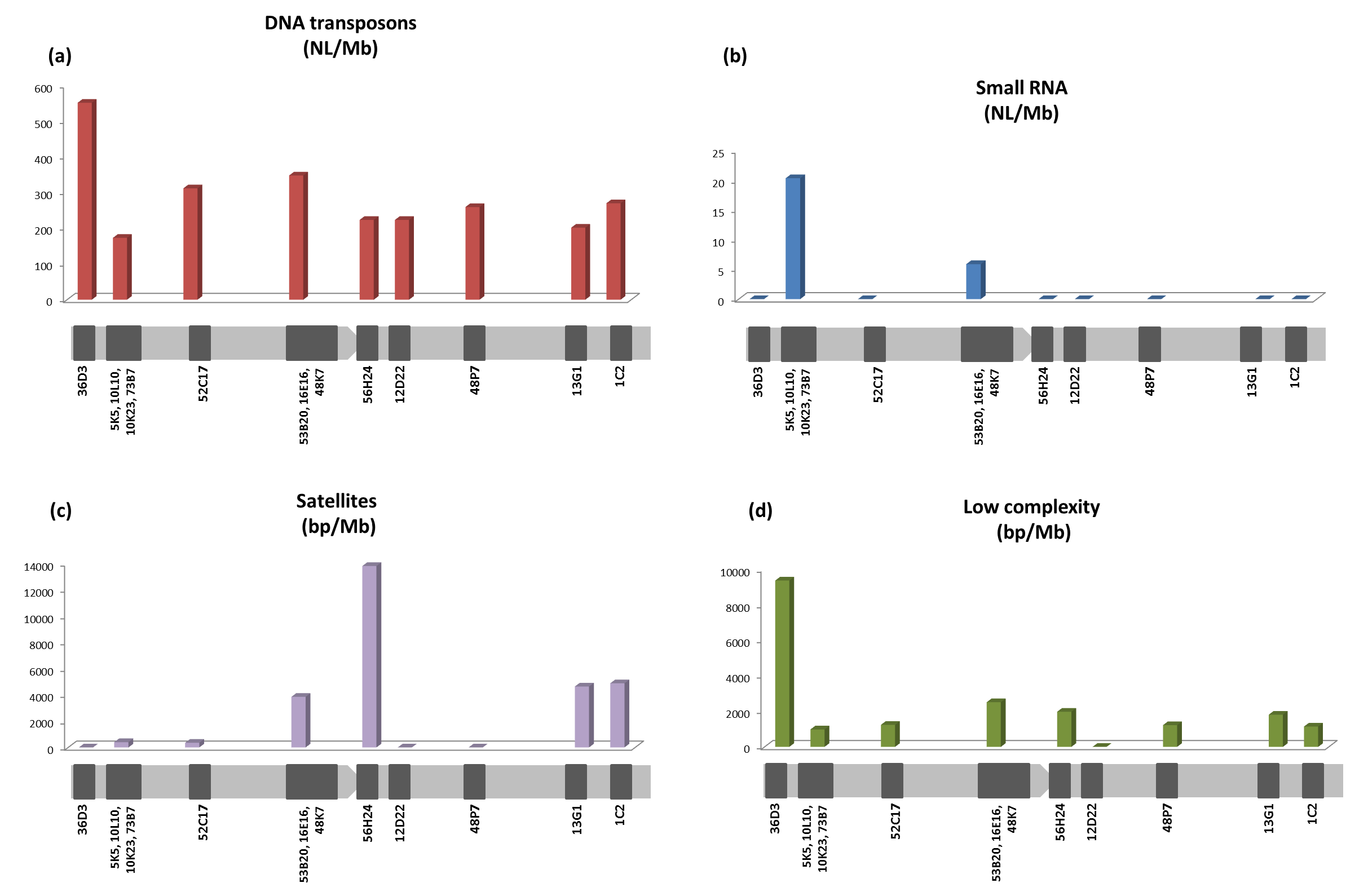

Supplement: Supplementary file 1 [file ijms-20-05111-s001.zip › ijms-615179-final sup/Figure S9.png]
